# Satisfaction of Basic Psychological Needs as Predictors of Motivation towards Physical Education in Primary Education: Influence of Gender and Physical Self-Concept

**DOI:** 10.3390/ijerph20247186

**Published:** 2023-12-15

**Authors:** Marta Prado-Botana, Miriam Carretero-García, Lara Varela-Garrote, Raúl Fraguela-Vale

**Affiliations:** Specific Didactics Department, Research and Diagnostic Methods in Education, Education Faculty, University of A Coruña, 15001 Coruña, Spain; m.prado1@udc.es (M.P.-B.); miriam.carretero@udc.es (M.C.-G.); lara.varela@udc.es (L.V.-G.)

**Keywords:** self-determination theory, basic psychological needs, motivation, primary education, physical education, gender, self-concept form 5, PBN scale, PLOC scale

## Abstract

The aims of this study are as follows: (a) to determine the level of physical self-concept, satisfaction with basic psychological needs (BNP), and motivation towards physical education (PE) among primary education school students; (b) to analyze the correlations between the different variables; to (c) identify gender differences in the studied variables; and (d) to examine the capacity of BPN, physical self-concept, and gender as predictors of different types of motivation towards PE. The sample comprises 474 primary school students (average age = 10.58; SD = 0.626) from four educational centers in A Coruña, Spain. A multivariable linear regression analysis was conducted to determine whether independent variables of BPN, gender, and physical self-concept can predict different types of motivation towards PE. The results show that satisfaction with the BPN of autonomy is significantly lower than the other two variables. Moreover, there is a positive and significant correlation between physical self-concept and satisfaction with BPN, as well as with intrinsic, identified, and introjected motivations. Boys outperform girls in physical self-concept, satisfaction with competence and socialization BPNs, and introjected, external, and amotivation motivations. The main conclusion is that BPNs solely forecast the most self-determined motivations (intrinsic and identified), have a restricted influence on introjected motivations, and do not predict external regulation or amotivation. Neither gender nor physical self-concept significantly contributes to predicting any motivation towards PE at these ages.

## 1. Introduction

Self-determination theory (SDT) was developed at the end of the last century and focuses on studying human behavior and personality development [1]. SDT is a broad framework of motivation and personality helpful in comprehending the determinants that influence psychological well-being and motivation in individuals. This is of great relevance generally and specifically applicable to the study of educational settings [2]. The SDT is a macro-theory composed multiple mini-theories, among them the basic psychological needs theory (BPN) [3]. These BPNs are autonomy, competence, and relatedness. They are considered universal, innate, and crucial, and it is necessary to fulfil these needs to have adequate health and functionality. The BPN theory [1] focuses on the relationship between the level of satisfaction of previous needs and the degree of individual well-being. This satisfaction or frustration of the needs directly depends on the individual, timing, context, and interpersonal relationships and applies to all areas of life. The need for autonomy is linked to the individual’s perception that they can do what they intend to do by themselves rather than relying on external factors [4], creating a causal locus for the person in their activities. Perceiving autonomy positively orients the needs for competence and relatedness while simultaneously predicting self-determined motivation positively [5]. The second need is competence, which involves an individual feeling capable of performing activities of variable levels of complexity. This need is highly predictive of intrinsic motivation, positively associated with adequate stress regulation, self-esteem and well-being and negatively associated with anxiety, low self-esteem, or depression [6,7]. The third need, relation, relates to the level at which individuals feel they belong to a collective, feeling connected to others in their social context [8]. Basic psychological needs are strongly correlated, serving as psychological mediators that influence motivation in various personal contexts.

Regarding the role of motivation within the SDT, it is structured on a continuum of multiple motivation levels, ranging from the least to the most self-determined behavior. The least self-determined is amotivation, where the motivation is null, and there is no intention to act. It describes a state in which the person is not motivated towards that behavior: they do not give value to it, do not get a reward, or do not give meaning to the action they have to perform. Next is extrinsic motivation, which is triggered in the individuals by external factors and exhibits multiple degrees: external regulation, which is promoted by external rewards or incentives; the introjected regulation, caused by avoiding feelings of guilt; the identified regulation, wherein individuals are interested in what they do because it aligns with their values; and the integrated regulation, whereby people internalize the process and carries it out by their own choice even if prompted by external factors, leading to increased persistence. This type of regulation (integrated) represents the highest level of determination within extrinsic motivation. It is mainly found in adults, so it does not appear in scales aimed at childhood and adolescence, as personality develops at this age, and it is not easy to develop this type of motivation [8]. Finally, the most self-determined motivation, intrinsic motivation, is found when the subject feels comfortable and takes pleasure in performing an activity for its own sake. This type of motivation is characterized as the highest level of personal motivation [9].

SDT has been proposed as one of the most significant theories for explaining motivation towards physical activity in sports and physical education (PE) [10,11]. Suppose the SDT approach [12] was applied in PE. In this case, it is expected that student engagement in meaningful, interesting, relevant, and enjoyable activities would satisfy their BPN and enhance their autonomous motivation, resulting in greater interest and increased transfer of learning outside the classroom. As a result, students would be more likely to increase their healthy behaviors during leisure time. Self-determination in physical education has three components: affective, emotional, and cognitive components. It involves perceiving the subject as enjoyable, fulfilling, worthless, and even humiliating, depending on the sensations and emotions experienced in it [13]. These aspects are affected by multiple factors, including the relationship between peers in the classroom, the type of session, the teaching style, the methodology, or the content being taught [14]. The problem lies in comprehending the teaching strategies required to enhance pupils’ motivation to participate in PE at school and to apply what they have learned in the classroom to their everyday lives [15].

Numerous research instruments are accessible for the application of SDT to the context of PE. One of these tools is the Spanish edition of the Basic Psychological Needs in Exercise Scale [15,16], which analyzes satisfaction of the BPN within the context of physical activity and sport. On this scale, the analysis focuses on the three dimensions: perception of autonomy, competence, and relatedness in PE. Positive scores in all three BPNs are expected to increase autonomous motivation [16,17,18,19,20,21]. This type of self-determined motivation among PE leads to increased enjoyment and satisfaction in classes, highlights the significance and usefulness of the subject, and positively enhances the learning outcomes in their lifestyle [22,23].

Satisfaction with the need for competence in PE is correlated with students’ general perception of competence [24]. This perception of competence is reflected in leisure physical activity: if the students feel proficient in PE, they will also do so in their motor practices outside the school. However, autonomy and relatedness needs have a more specific aspect and do not transfer as directly to contexts beyond PE [25]. To satisfy the needs for autonomy and competence, Carron et al. [26] suggest involving students in the cognitive development of tasks, ensuring that they are challenging, encouraging active participation, and varying in their nature. To improve the three BPNs, Niemiec and Ryan [27] propose teaching strategies that include providing different approaches in class to promote autonomy, giving feedback on competence and encouraging respect and care within the student group to foster relatedness.

To measure motivation in physical education among the age range studied, the Perceived Locus Of Causality Scale in PE (PLOC scale) is a suitable tool for assessing motivation in physical education among both boys [28,29]. Specifically, an adaptation of this scale for PE sessions, conducted by Moreno et al. [30] in the Spanish context, has been utilized in multiple contexts of primary and secondary education and was further developed by Moreno et al. [28]. Several studies have identified effective strategies to increase intrinsic motivation in students, such as teacher motivation of pupils in their classes, using alternative pedagogical models (e.g., student-centered or play-based approaches), and creating learning environments that develop motor skills. These approaches should be prioritized to improve intrinsic motivation [31,32,33].

Self-concept is a multidimensional term comprising various contexts or aspects of individuals, such as academic or occupational, social, emotional, familial, and physical [34]. In this study, we analyze the extensively used AF5 scale [35] to measure it in the context of primary education [36]. Physical self-concept refers to an individual’s holistic perception of their body, including their perception of motor skills, external appearance, and level of physical well-being [37]. The construct has been studied in different contexts. It exhibits a positive correlation with physical exercise and sports [18,38,39,40,41], along with better academic performance during physical education sessions [42] and increased self-determined motivations, among other factors [43].

The relationship between physical self-concept and motivation requires further research, as existing results are inconclusive. Although a connection between motivation and self-concept development has been identified, multiple studies have suggested that this connection is complex and that further research is needed [44]. Children with a positive physical self-concept show higher levels of intrinsic and extrinsic motivation towards physical education. Positive body perception and physical skills may foster intrinsic motivation by making physical activities more attractive and satisfying. On the other hand, a negative physical self-concept may affect extrinsic motivation, as children may be more dependent on external factors to participate in physical activities.

The current study investigates the relationship between the BPN, motivation towards physical education, and physical self-concept. Previous studies conducted on adolescents aged between 12 and 17 have found that fulfilling the needs of competence, autonomy, and relatedness results in self-determined motivations [5,13,45], and that the physical self-concept exhibits a positive correlation with self-determined motivations such as intrinsic motivation and identified regulation, as well as with introjected regulation. However, no correlation was found between the BPN and external regulation and amotivation [7]. However, additional information is required to comprehend the association between the BPN and motivation towards PE during primary education, particularly in preadolescent cohorts.

Gender is an essential factor when studying motivation towards PE in the early years. Previous research has highlighted a significant gender gap in PE motor tasks [46,47]. In general, pupils show discontentment towards the detachment of physical education from their everyday physical activity or motor interests. This feeling is intensified in females [48], who exhibit less activity during the lessons, highlighting the need to address gender disparities in PE.

Research into the relationship between gender and satisfaction of BPN in PE classes has mainly focused on secondary school students. Some studies suggest that boys display higher satisfaction levels concerning competence and socialization [49,50]. However, research conducted by Moreno-Casado et al. [15] discovered that girls experience greater satisfaction in the need for socialization, along with lower levels of frustration and boredom. The authors attribute these findings to using interpersonal methodological styles that focus on gender differences among students. This could be supported by research such as Monzó et al. [51], a study based on a didactic intervention where girls increased their values for al BPN. Regarding autonomy, statistically significant differences are also evident, with girls scoring lower than boys [49,52].

The high dropout rates from physical activity among females have generated interest [53,54] due to their apparent correlation with a lack of motivation towards PE [55]. Females demonstrate higher levels of amotivation towards PE classes compared to males. They also achieve lower scores on intrinsic motivation and introjected regulation, which some authors associate with reduced perceptions of competence and less interest in tasks and activities carried out in class [52,55,56,57]. However, other studies demonstrate no gender disparities in motivation, suggesting that intrinsic motivation and identified regulation are prevalent among both genders [53,58,59].

Another variable to consider in gender analysis, as pointed out earlier, is physical self-concept, which relates to the early age motivation and satisfaction of BPN. Research on adolescents has shown that physical self-concept shows significant gender differences, with boys achieving higher levels on this variable and girls showing a more negative perception of their body image [60,61].

The novelty of this research lies in the age bracket that provides the information. As previously stated in the introduction, we have insufficient details on the satisfaction of BPN in PE. Despite an increasing interest in this stage of education, there is still little work on the relationship between PBN and motivation for physical education in primary School. Understanding what occurs at this stage is crucial for comprehending the impact of puberty when it arrives during the secondary education phase.

A critical contribution of this study is its role in the discussion on the correlation between BPNs and various forms of motivation towards early PE. Certain studies have identified correlations between BPNs and all kinds of motivation, with favorable connections to more self-determined motivations and negative relationships towards less self-determined ones. However, research in populations under 12 is limited, and the results are inconclusive. An additional tendency indicates that this correlation is exclusive to BPNs and intrinsic and identified motivation and not to other motivators. This investigation aims to contribute new data to this ongoing debate.

Increasing understanding of the factors determining motivation towards PE at the end of primary school can help educational institutions engage students in the subject and promote healthy lifestyles from an early age. In the change of educational stage from primary to secondary education, the students need a teaching accompaniment that takes into account their physical and psychological evolution and that allows them to maintain the levels of motivation towards PE regardless of gender. Studying the last stage of primary education is vital to comprehending the initial phase of this transition process, where motivation towards PE has not decreased significantly yet, and gender differences are still moderate. The principal objectives of this study are as follows: (a) to determine the level of physical self-concept, satisfaction with BPN, and motivation towards PE (all variables under study) among primary education school students; (b) to analyze the existence of correlations between the different variables; (c) to identify gender differences in the studied variables; and (d) to examine the capacity of BPN, physical self-concept, and gender as predictors of different types of motivation towards PE.

Working hypotheses were developed based on the study’s findings and the research objectives. As previously stated, the information available primarily stems from studies conducted during the secondary education stage (adolescence) and less so during the primary education stage (preadolescence). Consequently, the hypotheses are more applicable to the reality of adolescents. It is expected that there may be slight variations from the patterns specified in the hypotheses, as the age groups being examined are not precisely identical. The study’s objectives led to the following hypotheses: (1) In the primary education stage, high values will be attributed to physical self-concept, satisfaction with BPN, and the most-determined motivation (intrinsic and identified), whereas the less self-motivated regulations will only have moderate values. (2) It is anticipated that all variables will have a positive correlation to one another, which includes physical self-concept, satisfaction with BPN, and more self-regulated motivations (intrinsic and identified). On the contrary, introjected and external regulations, as well as amotivation, are predicted to manifest negative correlations towards the above-mentioned variables. (3) Gender differences are expected in all studied variables, with males expressing higher values of self-concept and satisfaction with BPN and more self-determined motivations (intrinsic and identified). At the same time and expected to females score higher in the remaining categories of motivation (introjected, external and amotivation). (4) The hypothesis states that the independent variables (satisfaction with BPN, physical self-concept) will positively predict the more self-determined motivations (intrinsic and identified) and negatively predict introjected, external, and amotivation. Additionally, it is anticipated that gender will be a relevant predictor of motivation.

## 2. Materials and Methods

### 2.1. Design

A quantitative methodology and non-experimental cross-sectional correlational design was carried out.

### 2.2. Participants

A convenience non-probability sampling method was used for the study based on the accessibility of the educational centers.

The sample comprised 474 primary education students from four centers in A Coruña, Spain. The average age of the participants was 10.58 years (SD = 0.626), with a gender distribution among 49.8% of girls and 50.2% of boys.

### 2.3. Measurements

Student satisfaction with physical education was measured using a survey technique with the questionnaire as instrument. Three previously validated scales used in most of the research on physical education were used.

Basic Psychological Needs in Exercise Scale: The Spanish version of the scale, adapted for physics education, was used by Moreno et al. [16]. Initially designed by Vlachopoulos and Michailidou [17], the survey comprises 12 items grouped into three dimensions: autonomy, competence, and relation (four items per group). Every item has an original statement, “In my PE classes”, and they were evaluated on a Lickert Scale ranging from 1 (totally disagree) to 5 (totally agree). The McDonald’s Omega obtained for each of the three dimensions were as follows: autonomy, 0.77; competence, 0.71; and relatedness, 0.85.

Perceived Locus of Causality (PLOC): Designed by Goudas et al. [62] and translated into Spanish by Moreno et al. [30], it was used to determine the types of motivation perceived by the students. The survey is composed of 20 items (4 for each factor) and headed by the statement “I participate in this physical education class….”, evaluated on a Lickert Scale ranging from 1 to 5. Reliability coefficients (McDonald’s Omega) were as follows: intrinsic regulation, 0.82; identified regulation, 0.74; introjected regulation, 0.71; external regulation, 0.72; and amotivation, 0.68.

Self-concept form 5 (physical dimension): Designed by Garcia and Musitu [35], this scale rages in 30 items the five great domains of the self-concept: academic, social, emotional, familial, and physical. In the present study, only the physical dimension items were used (e.g., “I take good care of my physical health”, “I am good at sports”). They are evaluated on a scale from 1 (totally disagree) to 99 (totally agree). McDonald’s Omega coefficient for physical self-concept was 0.83.

### 2.4. Procedure

Data collection was carried out between April and September 2023. Initially, contact was made with educational institutions, informing them of the study’s objectives and procedures. Once the schools agreed to participate, students were informed about the study’s aims, and informed consent was provided by their families, with participation limited to those with authorization from their parents or legal custodians. The online questionnaire was provided to students through their Virtual Classroom, to be completed on their computers during instructional time, with at least one member of the research team present in the classroom to explain the procedure and clarify any student inquiries.

### 2.5. Data Analysis

To conduct data analysis, the statistical software program SPSS 28.0. and G*Power 3.1 were employed. The analysis included descriptive statistics, bivariate correlations, mean comparisons, and multiple stepwise linear regression.

To accomplish the first objective, measurements with standard deviations were calculated for gender, self-concept, basic psychological needs, and motivation towards PE. To determine distinctions between them, paired comparisons were conducted using the Student’s *t*-test for related samples.

The correlations between the variables (second objective) were examined using the Pearson correlation test.

Gender comparison between the different variables (third objective) was analyzed using Student’s independent samples *t*-test. Effect size was calculated to quantify the magnitude of the difference between the averages of the two groups.

To analyze the relationship between the BPN and different types of motivation (fourth objective), we conducted a multiple linear regression analysis to determine if the BPN predicts the types of motivation. Each type of motivation serves as a dependent variable, and the three BPNs serve as independent variables. As stated in the theoretical framework, gender and physical self-concept are potentially influential factors on motivation and thus were used as independent variables in the analysis. The forward stepwise selection procedure was applied for variable selection. Due to the length requirements for presenting all regression models, the results section presents the resulting models of each type of motivation (last step of forward introduction) in the order they were introduced.

## 3. Results

According to Table 1, the participants have a moderately high self-concept, scoring over 70 on a scale where the maximum value is 99.

To compare the scores obtained on the satisfaction of the BPN and motivation scales, pairwise comparisons of variables were made using the related samples Student’s *t*-test. According to the results, the autonomy BPN scores were significantly lower than the competence and relatedness (*p* < 0.001). In contrast, there were no significant differences between the latter two (*p* > 0.05). The autonomy’s value can be considered moderate since it slightly exceeds the scale’s central point. In comparison, competence and relatedness obtain higher scores, surpassing four points.

Significant differences exist between all types of motivation. The majority demonstrate *p* < 0.001 level significance, except for the most self-determined regulations pair (intrinsic vs. identified), which reaches *p* < 0.05 level. A higher number of students have the two most self-determined motivations, with reduced appreciation as they become less self-regulated.

Table 2 presents the correlations among the examined variables. A significant and positive association is detected between physical self-perception, satisfaction with the three BPNs and intrinsic, identified, and introjected motivations. However, no significant correlation is observed between physical self-concept and extrinsic motivation and amotivation. BPNs are closely linked to each other and have more self-determined types of regulation (intrinsic, identified, and introjected). However, no relationship was found between external regulation and motivation, except for the case of satisfaction of BPN for competence, which moderately correlates with external regulation.

Regarding the different categories of motivation, a strong and positive relationship exists between those categories that are more closely related. Furthermore, increased levels of intrinsic motivation result in elevated levels of identified and introjected motivation, among others.

The only significant negative relationship exists between intrinsic motivation and amotivation.

As noted in the introduction, gender has been identified as a prominent factor in research on motivation towards PE [61,63]. Using the independent samples Student’s *t*-test, we examine whether there are any notable differences between the assessments of the studied variables among boys and girls. Table 3 displays that male participants exhibit higher scores in some areas. Specifically, physical self-concept and satisfaction of BPN for competence show highly significant differences, while external, introjected, and amotivation do not. The significance of these differences should be interpreted with caution, however, as the effect size is small [64] since all the effects are smaller than 0.320. No differences were found in the autonomy BPN or the most self-determined regulations (intrinsic and identified).

The regression analyses show in Table 4 suggest that the BPNs solely work as a dependable prognosticator of intrinsic and identified motivation (clarifying 43% and 31% of the variance, respectively). Autonomy BPN is the most influential predictor of both regulations, with gender and physical self-concept excluded from the model.

Competence and autonomy BPN, along with gender, are predictors of introjected regulation. However, the explained variance percentage (12%) and the change in R^2^ and F recommend not using this model for predicting introjected motivation. Instead, other factors with a more significant influence on the dependent variable should be explored.

This phenomenon is also observed with extrinsic regulation and amotivation in which all variables, except gender, are excluded from the model except for gender, whose contribution to predicting both variables is minimal (1% of the explained variance).

Satisfaction with the BPN requirement indicates intrinsic and identified motivation towards PE, but not introjected, external, or amotivation.

The relevance of gender as a predictor for motivation towards physical education is minimal, as it is excluded from the models that predict intrinsic and identified motivations based on the BPN. When it is included as a predictor, its contribution towards explaining the dependent variable’s variance is minimal.

Physical self-concept is an inconsistent predictor of different types of motivation. It has not been included in any of the regression models, thereby failing to account for the variability of the different dependent variables.

## 4. Discussion

For a better understanding of the discussion, the following is structured around the four objectives of the study.

### 4.1. Levels of Physical Self-Concept, Satisfaction with the BPN, and Motivation towards PE

Regarding the first objective of the study, the descriptive data of the various types of motivation towards physical education favor the subject. This implies that in general, the participants are intrinsically motivated and highly enjoy the activities they participate in during their classes, in line with Navarro-Patón et al. [65]. The least satisfied BPN is autonomy, as both competence and relatedness achieve high values, surpassing 4 points on a scale of 1 to 5. Moreover, physical self-concept values surpass 70% of the maximal achievable score and are considered high. The initial hypothesis is practically confirmed, as we anticipated physical self-concept levels, satisfaction with the BPN, and more self-determined motivations (intrinsic and identified regulations) would exhibit high values. In contrast, less self-motivated regulations would show moderate values. However, only the satisfaction of the autonomy BPN deviates from our hypothesis by presenting lower values.

Despite educational recommendations promoting competency-based work and student-centered methodologies [27,66], the study participants report a reduced level of satisfaction regarding autonomy in the PE subject. They note that their activities do not align with their interests and desires, nor the manner they would prefer to execute them, and they are given limited opportunities to choose how to perform them. The study indicates that physical education’s teaching methodology still centers on the instructor, and primary school pupils in the final stage have restricted chances to make basic decisions and take initiative in their exercises. This aligns with multiple studies, such as Burgueño et al. [67] and Gil-Arias et al. [68], among others. Students in PE classes can develop self-determined motivation if they feel involved in decisions about the subject matter and feel positively connected with their peers. [16]. Beyond theoretical knowledge of PE, teachers must acknowledge the impact of their professional performance on students. The structure of the subject, the teacher’s conduct during classes, and the atmosphere they generate will significantly affect students’ motivation towards the subject [69,70,71].

### 4.2. Correlation of Physical Self-Concept, BPN, and Motivation towards PE

In line with previous research [43,46,72], the correlation study (objective 2 of the study) has found a significant and positive association between physical self-concept and satisfaction with BPN, as well as these constructs with more self-determined motivations towards PE (intrinsic, identified, and introjected). Our second hypothesis is only partly fulfilled, as we predicted the previously described correlation but also expected a contradictory outcome (negative correlation between physical self-concept, BPN, and intrinsic motivation with less self-determined motivations). However, this relationship appears uneven since, except for one case (the relationship between intrinsic motivation and amotivation), high levels of physical self-concept and satisfaction with BPN do not negatively correlate with extrinsic motivation or amotivation.

In this manner, high levels of physical self-concept and satisfaction with BPN would contribute to more self-determined motivations. However, they would not “protect” against high levels of extrinsic motivation and amotivation. These results are not contradictory, as specific high valuations in a BPN, such as competence, are compatible with high levels of extrinsic motivation. In this example, the teaching reinforcement of the student’s positive motor responses to their proposals will surely help motivate them towards the subject, even if it is in an extrinsic way (through teacher approval-reinforcement).

### 4.3. Gender Analysis: Physical Self-Concept, BPN, and Motivation towards PE

When comparing boys and girls (objective 3), the study found that boys have higher levels of physical self-concept and satisfaction of BPN for competence and, to a lesser extent, relatedness than girls. These results align with previous research conducted on preadolescents and adolescents [49,50]. Regarding motivation towards PE, the study revealed that boys and girls equally value self-determined regulations, both intrinsic and identified. These results reinforce that the existing research does not observe gender differences in intrinsic and identified motivations towards PE during the early stages [53,58,59]. We reiterate the earlier findings regarding the lack of the BPN for autonomy, exhibiting lower scores than the other BPN. The boys and girls agree that their need for autonomy needs to be more satisfactorily fulfilled in this aspect. Considering the reduced effect size of gender on all examined variables, this study’s results align with research indicating moderate or non-existent differences between boys and girls in terms of satisfaction with PBN and motivations towards PE during primary education [49].

The third hypothesis of the study, which anticipated gender disparities in all examined variables, has been refuted. This non-compliance with the hypothesis enables us to identify a turning point in the influence of gender on satisfaction with BPN and motivation between primary and secondary education or between pre-adolescence and adolescence.

### 4.4. BPN, Physical Self-Concept, and Gender as Predictors of Motivation towards PE

The research examining the capability of BPN, physical self-concept, and gender to predict the different types of motivation towards PE classes (objective 4) demonstrates that BPNs are only able to forecast self-determined regulations (intrinsic and identified) and to moderately predict introjected regulation, and they do not predict external regulation or amotivation. These findings partially correspond to previous research, which revealed that satisfaction with BPN directly predicted motivation towards PE in the case of more determined regulations and inversely in the case of less determined ones [7].

The correlation analysis shows that the impact of BPN on motivation towards PE is not symmetrical and is further affirmed by the regression analysis. This impact primarily affects more self-determined regulations. The relationship (especially between BPN and intrinsic motivation) has been extensively examined in adult and adolescent contexts. However, research on SDT in primary education is less frequent.

Gender is not a significant predictor of motivation towards PE in this study. However, gender differences exist, with children of different genders displaying distinct values for the relevant variables. Nonetheless, the consistent regression models for intrinsic and identified regulation behave similarly; therefore, they apply to both groups.

Like gender, self-concept is not a predictor variable for motivation towards education. However, it is highly correlated with all the variables studied, so its role in BPN satisfaction and motivation towards PE needs to be further explored [72].

The study’s findings partly support the first part of the fourth hypothesis, as BPN positively predict more self-determined motivations (intrinsic and identified) towards PE. However, there is no negative prediction regarding introjected, external, and amotivation. The expectations concerning intrinsic motivation and gender are also unfulfilled as they do not hold any importance in predicting motivations towards PE in primary education.

## 5. Conclusions

The study’s main findings suggest that levels of satisfaction of competence and relatedness BPN in PE are high. However, satisfaction with the need for autonomy is significantly lower. This implies that implementing the methodological change, whereby students are placed at the center of the learning process, is not being effectively applied in this subject.

Moreover, a significant and positive correlation has been noticed between physical self-concept, basic psychological needs (PBN), and the most self-determined forms of motivation, including intrinsic, identified, and introjected motivations. The only significant and negative relationship detected was between intrinsic motivation and amotivation.

The influence of gender on physical self-concept, satisfaction with BPN, and motivation towards PE is moderate in primary education in contrast to studies in secondary education, where this impact is considerably stronger.

It has been discovered that satisfaction with BPN is a strong predictor of intrinsic and identified motivation, which reduces its influence on introjected regulation, with significantly less impact and only with competence and autonomy BPN. Satisfaction with BPN does not predict less self-determined motivation as might be expected. Gender and physical self-concept are not significant predictors of motivation towards PE at these ages.

The results emphasize the importance of satisfying BPN for enhancing intrinsic and identified motivation. Additionally, they indicate an urgent requirement for additional studies to identify the factors that predict external regulation and amotivation to PE during preadolescence (primary education stage).

### 5.1. Practical Implications

The main practical implication of this work is related to students’ need for autonomy in PE classes. The first predictor of the two most self-determined regulations is the autonomy BPN, which is precisely the one satisfied to a lesser extent in PE sessions. These data suggest the need for PE teachers to follow the recommendations of current curricula, which urge them to use more experiential and student-based methodologies in their teaching, especially in the final years of primary school. Autonomy BPN needs to be met by PE, which justifies a change of approach to the subject. It is also the factor that best predicts intrinsic and identified motivations, which are the most desirable for achieving a positive and lasting link with PE and favor pupils enjoying the benefits of practicing PA in the future.

### 5.2. Limitations of the Study

Among the main limitations of this research, we highlight the following:

- The exploratory nature of this research and the lack of references to contrast whether the existing models are applicable to the primary school stage make this research a necessary first step in exploring the variables that determine motivation towards the subject of PE. However, it remains to be seen whether the existing models of PE towards adolescents and young adults are applicable to pre-adolescents and, if not, to construct a specific model for this stage of life.
- The study focuses only on the satisfaction of BPN and not on the frustration of these needs, which may be relevant to understanding the relationship between NPB and motivation towards PE.

### 5.3. Future Lines of Investigation

- To include new variables in the analysis of the factors that influence motivations towards PE, to compare the validity of existing models of motivation towards the subject and, if necessary, to construct a specific model for the pre-adolescent population (last years of primary education). Among these variables, BPN frustration should be relevant to understanding the relationship between BPN and motivation towards PE.
- Increase the diversity of the sample by including centers in both urban and rural areas.
- Develop new tools specifically for primary school pupils to assess the satisfaction and frustration of BPNs in PE.
- Use mixed-methods research designs to give learners a voice in interpreting the results of the BPN and PE motivation scales (sequential explanatory model).

## Figures and Tables

**Table 1:** Descriptives of physical self-concept and BPN with motivation towards PE (n = 474).

Variables		M	SD
Self-Concept Form 5	Physical Self-concept	71.04	21.58
PBN scale	Autonomy	3.38	0.90
	Competence	4.20	0.69
	Relatedness	4.24	0.85
PLOC scale	Intrinsic R.	4.20	0.86
	Identified R.	4.27	0.78
	Introjected R.	3.24	1.04
	External R.	2.95	1.09
	Amotivation	2.04	1.00

Note: M = mean; SD = standard deviation.

**Table 2:** Correlations of physical self-concept, BPN, and motivation towards PE subject.

	A	C	R	IT	ID	IN	EX	AM
PSC	0.18 ***	0.51 ***	0.35 ***	0.26 ***	0.24 ***	0.15 ***	−0.031	−0.11
A		0.47 ***	0.40 ***	0.56 ***	0.45 ***	0.29 ***	0.05	−0.02
C			0.49 ***	0.50 ***	0.45 ***	0.29 ***	0.09 *	−0.03
R				0.49 ***	0.41 ***	0.24 ***	0.03	−0.06
IT					0.70 ***	0.42 ***	0.06	−0.15 ***
ID						0.53 ***	0.19 ***	−0.04
IN							0.52 ***	0.29 ***
EX								0.44 ***

Note: * *p* < 0.05, *** *p* < 0.001. PSC = physical self-concept; A = PBN autonomy; C = PBN competence; R = PBN relatedness; IT = intrinsic regulation; ID = identified regulation; IN = introjected regulation; EX = external regulation; AM = amotivation.

**Table 3:** Physical self-concept, BPNs, and motivation towards the subject. Comparative between boys and girls. Independent samples *t*-test (n = 474).

Variables		Gender					
		Boys (n = 238)		Girls (n = 236)		T	ρ
		M	SD	M	SD		
S-C Form 5	Physical Self-concept	74.42	23.56	67.63	18.87	−3.465 ***	0.319
PBN Scale	Autonomy	3.33	0.92	3.44	0.88	1.307	0.120
	Competence	4.31	0.64	4.09	0.72	−3.611 ***	0.332
	Relatedness	4.33	0.80	4.14	0.90	−2.432 *	0.223
PLOC Scale	Intrinsic R.	4.26	0.83	4.13	0.89	−1.712	0.157
	Identified R.	4.32	0.77	4.21	0.79	−1.551	0.143
	Introjected R.	3.35	1.06	3.13	1.00	−2.325 *	0.214
	External R.	3.10	1.08	2.79	1.08	−3.021 **	0.278
	Amotivation	2.15	1.00	1.93	0.99	−2.376 *	0.218

Note: * *p* < 0.05, ** *p* < 0.01, *** *p* < 0.001. M = mean; SD = standard deviation. T = t-value (Independent samples *t*-test); ρ = rho-value (effect size).

**Table 4:** Multiple linear regression coefficients considering types of motivation as dependent variables.

DV	IV	R^2^	ΔR^2^	ΔF	β
Intrinsic R.		0.43	0.03	26.90 ***	
	PBN Autonomy				0.36 ***
	PBN Relatedness				0.23 ***
	PBN Competence				0.22 ***
Identified R.		0.31	0.27	18.51 ***	
	PBN Autonomy				0.26 ***
	PBN Competence				0.23 ***
	PBN Relatedness				0.19 ***
Introjected R.		0.12	0.00	4.07 *	
	PBN Competence				0.18 ***
	PBN Autonomy				0.21 ***
	Gender				0.08 *
External		0.01	0.01	9.1 **	
	Gender				0.13 **
Amotivation		0.01	0.01	5.64 *	
	Gender				0.10 *

Note: * *p* < 0.05, ** *p* < 0.01, *** *p* < 0.001. DV = dependent variables; IV = independent variables; R^2^ = R Square; ΔR^2^ = R Square change; ΔF = F change; β = Standardized Beta Coefficients.

## Data Availability

The datasets used during the current study are available from the corresponding author upon reasonable request.
